# Retrospective epidemiological study of canine epilepsy in Japan using the International Veterinary Epilepsy Task Force classification 2015 (2003–2013): etiological distribution, risk factors, survival time, and lifespan

**DOI:** 10.1186/s12917-016-0877-3

**Published:** 2016-11-09

**Authors:** Yuji Hamamoto, Daisuke Hasegawa, Shunta Mizoguchi, Yoshihiko Yu, Masae Wada, Takayuki Kuwabara, Aki Fujiwara-Igarashi, Michio Fujita

**Affiliations:** Department of Clinical Veterinary Medicine, Nippon Veterinary and Life Science University, 1-7-1 Kyonan-cho, Musashino-shi, 180-8602 Tokyo Japan

**Keywords:** Dogs, Epilepsy, Idiopathic epilepsy, Lifespan, Risk factor, Structural epilepsy, Survival time

## Abstract

**Background:**

Epilepsy is the most common neurological disease in veterinary practice. However, contrary to human medicine, epilepsy classification in veterinary medicine had not been clearly defined until recently. A number of reports on canine epilepsy have been published, reflecting in part updated proposals from the human epilepsy organization, the International League Against Epilepsy. In 2015, the International Veterinary Epilepsy Task Force (IVETF) published a consensus report on the classification and definition of canine epilepsy. The purpose of this retrospective study was to investigate the etiological distribution, survival time of dogs with idiopathic epilepsy (IdE) and structural epilepsy (StE), and risk factors for survival time, according to the recently published IVETF classification. We investigated canine cases with epilepsy that were referred to our teaching hospital in Japan during the past 10 years, and which encompassed a different breed population from Western countries.

**Results:**

A total of 358 dogs with epilepsy satisfied our etiological study criteria. Of these, 172 dogs (48 %) were classified as IdE and 76 dogs (21 %) as StE. Of these dogs, 100 dogs (consisting of 65 with IdE and 35 with StE) were included in our survival study. Median survival time from the initial epileptic seizure in dogs with IdE and StE was 10.4 and 4.5 years, respectively. Median lifespan of dogs with IdE and StE was 13.5 and 10.9 years, respectively. Multivariable analysis demonstrated that risk factors for survival time in IdE were high seizure frequency (≥0.3 seizures/month) and focal epileptic seizures.

**Conclusions:**

Focal epileptic seizures were identified as a risk factor for survival time in IdE. Clinicians should carefully differentiate seizure type as it is difficult to identify focal epileptic seizures. With good seizure control, dogs with IdE can survive for nearly the same lifespan as the general dog population. Our results using the IVETF classification are similar to previous studies, although some features were noted in our Japanese canine population (which was composed of mainly small-breed dogs), including a longer lifespan in dogs with epilepsy and a larger percentage of meningoencephalomyelitis of unknown origin in dogs with StE.

## Background

Epilepsy is a common chronic and functional brain disorder in dogs and humans that is characterized by recurrent epileptic seizures. In the veterinary field, classification and terminology of epilepsy in part reflects current proposals from the human epilepsy organization, the International League Against Epilepsy [1–4]. However, a consensus on classification and terminology in veterinary medicine had not until recently been agreed; diagnostic procedures of epilepsy are slightly different between humans and animals, therefore routine examinations in human medicine (e.g., electroencephalogram (EEG) or functional imaging) have limitations in veterinary medicine. In order to address this problem, the International Veterinary Epilepsy Task Force (IVETF) was recently organized from specialists of veterinary neurology and other neuroscientists. Accordingly, new consensus reports on canine epilepsy were published in 2015 [5–11].

According to the IVETF consensus, “epilepsy is defined as a brain disease characterized by an enduring predisposition to generate epileptic seizures” [5]. Consequently, IVETF etiologically classified canine epilepsy into idiopathic epilepsy (IdE), structural epilepsy (StE), and unknown cause [5]. Additionally, IdE is further divided into genetic epilepsy, suspected genetic epilepsy, and epilepsy of unknown cause. Classification of dogs into genetic or suspected genetic epilepsy requires genetic and/or family analysis.

The IVETF criteria for IdE diagnosis is described by a three-tier system [6]. The tier I confidence level describes a history of two or more unprovoked epileptic seizures occurring at least 24 h apart, with an age at epileptic seizure onset of between 6 months and 6 years, an unremarkable interictal physical and neurological examination, and no significant abnormalities on minimum data base (MDB) blood tests and urinalysis. The tier II confidence level describes unremarkable fasting and postprandial bile acids, brain magnetic resonance imaging (MRI), and cerebrospinal fluid (CSF) analysis. The tier III confidence level describes characteristic EEG abnormalities for seizure disorders. In addition, the IVETF consensus recommends performing MRI and CSF analysis in dogs with the following conditions: age of initial epileptic seizure onset <6 months or >6 years, neurological deficits, cluster seizures (CS) or status epilepticus (SE) at initial epileptic seizure onset, and cases previously diagnosed as presumptive IdE but showing single antiepileptic drug (AED) resistance.

Because the IVETF classification has only recently been defined, there have not yet been etiological or survival studies of canine epilepsy based on this classification system. Previous studies of lifespan in dogs with epilepsy have been reported using different (conventional) classifications. A recent study reported median lifespan to be 9.2, 5.8, and 7.6 years for dogs with IdE, StE, and epilepsy from all causes, respectively [12], with premature death due to epilepsy-related causes. Moreover, some studies have focused on CS [13, 14] and/or SE [15], and reported that dogs with frequent CS may be associated with euthanasia [13], while dogs with SE may have a short survival time [15].

Here, we retrospectively investigated the etiological distribution of canine cases with epilepsy, which had been referred to our teaching hospital in Japan (Tokyo) during the past 10 years (2003–2013). In this study, distribution of breeds in the canine population was different from Western countries. The purpose of our study was to classify dogs with epilepsy according to the recent IVETF classification in 2015, and to investigate survival time, lifespan, and risk factors influencing survival time in dogs with IdE and StE.

## Methods

Because this was a retrospective and survey study, ethics for animal use was not requested. Nevertheless, all owners of the dogs included in this study had agreed to use of their dogs’ data for academic education and studies, and had previously signed a consent form on the first presentation to the teaching hospital.

The present study consisted of two components: 1) a study of the etiological distribution at the time of epilepsy diagnosis; and 2) a survival study performed using a questionnaire survey, which included evaluating risk factors associated with survival.

### Definition and inclusion criteria

#### Definition and inclusion criteria of epilepsy

According to IVETF consensus [6], epilepsy was defined as cases with a history of at least two unprovoked epileptic seizures >24 h apart. Cases diagnosed or suspected of reactive seizures due to metabolic and/or toxic diseases such as hepatic encephalopathy, hypoglycemia, and electrolyte disturbances were excluded. Cases diagnosed or suspected of paroxysmal events such as cardiogenic or vagotonic syncope, narcolepsy, vestibular attack, and movement disorders by various diagnostic tests (including semiological videos) were also excluded.

#### Definition and inclusion criteria of idiopathic epilepsy

IdE was defined as dogs with epilepsy (defined above) having an age at initial epileptic seizure onset of between 6 months and 6 years, unremarkable interictal physical and neurological examinations, and no clinically significant abnormalities on blood tests and urinalysis. Although our blood tests did not completely match the MDB suggested by IVETF, most cases corresponded (i.e., complete blood count (CBC), sodium, potassium, chloride, calcium, phosphate, alanine aminotransferase, alkaline phosphatase, total bilirubin, urea, creatinine, total protein, albumin, glucose, cholesterol, and triglycerides), with the exception of bile acids and ammonia. This was because patients who showed reactive seizures from metabolic diseases and/or toxic diseases who were tested for fasting and postprandial bile acids and ammonia were excluded from this study as described above. Moreover, urinalysis data were not used as inclusion criteria for this study, because insufficient urinalysis had been performed in many cases. Furthermore, dogs that showed or were clearly suspected of AED-induced or postictal neurological abnormalities were included with careful and multiple evaluations by a neurologist (DH). Additionally, we classified dogs that met the criteria for tier I according to IVETF guidelines except for age at epileptic seizure onset and having normal MRI and CSF findings (i.e., tier II) as IdE. Exceptionally, dogs with interictal neurological deficits and/or abnormal MRI findings that were suspected as postictal brain damage were included as IdE under the following conditions: severe CS or SE cases showing limbic or focal T2-weighted or fluid attenuated inversion recovery (FLAIR) hyperintensities (images were obtained <10 weeks from the last seizure [6]), but no neurological deterioration (except for epileptic seizures) over a period of >1 year.

#### Definition and inclusion criteria of structural epilepsy

StE was defined as dogs with epilepsy having abnormal MRI and/or CSF findings, regardless of age at seizure onset. In addition, StE included cases clinically diagnosed with degenerative, anomalous, neoplastic, inflammatory, infectious, traumatic, and vascular diseases (DAMNIT-V or VITAMIND, except metabolic and toxic) by signalment, clinical course, and MRI/CSF findings. Degenerative disease was diagnosed by a young age at onset, a subacute to chronic progressive course, and symmetrical abnormal MRI findings. Anomalous disease was diagnosed by non- or less progressive neurological signs with structural forebrain anomalies by MRI (e.g., hydrocephalus, arachnoid cyst, and cortical dysplasia). Neoplastic disease was diagnosed by an old age at onset, an acute to chronic progressive course, and recognized intracranial mass formation by MRI (e.g., meningioma and glioma). Inflammatory disease (e.g., meningoencephalomyelitis of unknown origin (MUO)) was diagnosed by typical MRI and/or CSF findings, including titers for some agents. Traumatic disease was diagnosed by a history of head trauma with asymmetrical injury findings by MRI. Vascular disease was diagnosed by an acute onset, improved clinical course, and focal (regional) abnormal MRI findings including hemorrhages. Although some cases (those that had undergone surgery or postmortem necropsy) had a definitive diagnosis, most of these sub-classified categories were determined clinically (as described) without definitive (pathological, genetic, or serological) diagnosis.

#### Classification of seizure type and definition of terms

According to IVETF classification [5], seizure types were classified into focal epileptic seizures (FES), FES that evolved into generalized epileptic seizures (FEvG), and generalized epileptic seizures (GES). Seizure types were determined by semiological interviews with the owner (all cases) or recorded ictal video and/or EEG findings (cases were classified as FES or FEvG if focal spikes were found). Regarding specific seizure patterns, CS was defined as two or more epileptic seizures within 24 h, and as SE by a single epileptic seizure lasting more than 5 min, or two or more discrete epileptic seizures without complete recovery of consciousness.

The seizure frequency of each patient was acquired at two time-points; at the time of the first presentation at the teaching hospital as determined from medical records, and at the time of the last follow-up as determined from the questionnaire as a continuous variable of mean number of seizures per month (sz/month).

Survival time was defined as the time period from the initial epileptic seizure until the date of death or last follow-up. Lifespan was defined as the time period from the date of birth until the date of death or last follow-up.

### Magnetic resonance imaging and cerebrospinal fluid analysis

Although the MRI performed in this study did not adhere to the epilepsy-specific MRI protocol suggested in the IVETF proposal for the IdE tier II confidence level [6, 10], all MRI scans included T1-weighted, T2-weighted, FLAIR, and contrast-enhanced T1-weighted images in the transverse plane. Images were obtained using a 1.5-Tesla system [Visart® 1.5 Tesla, Toshiba Medical System, Tokyo, Japan (between April 2003 and October 2009)] or 3.0-Tesla system [Signa® HDxt 3.0T, GE Healthcare, Tokyo, Japan (between October 2009 and March 2013)]. Furthermore, in cases examined by 3.0-Tesla, 3D T1- (pre- and post-contrast) and T2-weighted images were obtained, with multiplanar reconstructions provided for review, as suggested in the epilepsy-specific MRI protocol [10]. All MR images were reviewed by a neurologist (DH). When CSF analysis was available, CSF tapping was performed via cisternal puncture following MRI, and analyzed by (at least) cell count, cytology, and protein measurement. Exceptionally, in some cases, MRI (using 0.4–1.0-Tesla systems) and CSF analysis were performed in other institutes before referral to our teaching hospital. Nevertheless, MRI included the sequences described above (except for 3D sequences), and the same CSF analyses were performed.

### Etiological distribution study and statistical analysis

Medical records from the Neurology and Neurosurgery units of the Veterinary Medical Teaching Hospital of Nippon Veterinary and Life Science University (Tokyo, Japan) were searched for dogs that had presented from April 2003 to March 2013 with a chief complaint of seizures. In general, our teaching hospital admits referral cases only. The following data were extracted: (1) breed; (2) body weight; (3) gender and neuter status; (4) age at first presentation; (5) age at initial seizure onset; (6) seizure type; (7) seizure frequency; (8) presence of CS or SE; (10) interictal neurological findings; (11) MRI/CSF findings; (12) number and type of AEDs; and (13) other treatments. After surveying all the cases, those with reactive seizures or paroxysmal events (non-epileptic seizures) were excluded, and dogs with epilepsy were classified into IdE and StE based on our inclusion criteria (described above). StE cases were further sub-classified into DAMNIT-V categories based on clinical diagnosis.

For statistical comparisons between IdE and StE, chi-squared tests were performed for categorical data (gender, neuter status, seizure type, CS/SE, multiple AED use) and Mann–Whitney *U* tests were performed for continuous data (body weight and seizure frequency at the time of first presentation). Values of *P* < 0.05 were considered significant. Statistical analyses were performed using the EZR 1.28 software package (Saitama Medical Center, Jichi Medical University, Saitama, Japan) [16].

### Survival study and statistical analysis

A standardized questionnaire was sent to referring veterinarians in March 2014. The questionnaire included current or changed seizure frequency since start of treatment, seizure type (if changed), status of treatment (type of AEDs and other treatments e.g., glucocorticoids, other immunosuppressive drugs, decompressive drugs), mortality status (i.e., dead or alive), and cause and time of death (if applicable). Dogs whose survival status (alive or dead) could be confirmed from questionnaire responses were included in the survival study.

The Kaplan–Meier method with log-rank test was used to estimate median survival time and median lifespan for all epilepsy cases (including IdE and StE), IdE, and StE (including sub-classified categories). Dogs alive at the time of follow-up were censored. Furthermore, the Kaplan–Meier method with log-rank test was used to estimate median survival time in all epilepsy cases, IdE, and StE cases stratified by body weight (median), gender, neuter status, seizure frequency at the last follow-up, presence of CS and SE, seizure type, and multiple AED use. In order to incorporate seizure frequency at the last follow-up into the log-rank test, it was divided for survival analysis into <0.3 and ≥0.3 sz/month. This baseline of 0.3 sz/month was decided from the ‘acceptable seizure frequency’ (less than one seizure in 3 months) suggested in the IVTEF proposal [9, 17]. To assess the risk factor for survival time in all epilepsy cases and IdE, the Cox proportional hazard model was employed using the forced-entry method. To avoid multicollinearity, independent variables were used in the model. All factors were entered into the Cox proportional hazard model. The variables with *P* values of <0.3 in the first analysis remained in the final model, and other variables were removed. The final Cox proportional hazard analysis was then performed within the remaining variables. Because the StE group consisted of a small number of cases that were heterogenous, we excluded StE cases from the Cox hazard analysis. The *P* value, hazard ratio (HR), and confidence interval (CI) were calculated. *P* values < 0.05 were considered significant. All survival analyses were performed using EZR.

## Results

### Etiological distribution at the time of epilepsy diagnosis

Of 19,193 dogs admitted to the hospital during the study period, 472 dogs had seizure events (including 5 dogs (1.0 %) with reactive seizures), and 358 dogs (1.87 %) satisfied the definition of epilepsy. Of these 358 dogs, the following diagnostic tests were performed at the time of diagnosis: CBC and serum biochemistry profile except bile acids and ammonia (*n* = 337), urinalysis (*n* = 12), MRI (*n* = 185), CSF analysis (*n* = 35), and EEG (*n* = 7). Additionally, 22 videos of epileptic seizure were acquired from owners. Consequently, 172 dogs (48.0; 0.90 % of total) were classified as IdE and 76 (21.2; 0.40 % of total) as StE. The remaining 110 dogs could not be classified into either group; of these, 80 dogs had an age at initial epileptic seizure of <6 months or >6 years, 60 dogs had neurological deficits, and none had both MRI and CSF analysis performed. Only one dog with an initial seizure onset at 6.8 years old was included in the IdE category because of normal MRI and CSF findings. Additionally, four dogs with postictal brain damage due to SE were judged as IdE because of >1-year follow-up. Clinical data for all dogs with epilepsy and the breed distribution in each category (all epilepsy cases, IdE, and StE) are provided in Tables 1 and 2, respectively.

**Table 1:** Clinical data at the time of diagnosis for dogs with epilepsy, idiopathic, and structural epilepsy

Variables	Number of dogs		
	Epilepsy (*n* = 358)	IdE (*n* = 172)	StE (*n* = 76)
Gender			
Male [Neuter]	193 (54 %) [71 (20 %)]	97 (56 %) [37 (22 %)]	34 (45 %) [14 (18 %)]
Female [Neuter]	165 (46 %) [79 (22 %)]	75 (44 %) [41 (24 %)]	42 (55 %) [16 (21 %)]
Body weight (kg)			
Median	6.0	6.2	5.9
Range	1.0–54.0	1.3–37.8	1.0–39.5
Age at initial seizure onset (years)			
Median	3.6	2.5	5.5
Range	0.1–14.4	0.5–6.8	0.2–13.9
Neurological			
Normal or unremarkable	117 (33 %)	170 (99 %)	24 (33 %)
Deficits	241 (67 %)	2 (1 %)	52 (67 %)
Seizure frequency at first presentation (sz/month)			
Median	2.0	1.2	4.0*
Range	0.1–37.0	0.1–37.0	0.1–30.0
Seizure type			
FES	93 (26 %)	52 (30 %)*	10 (13 %)
GES	190 (53 %)	78 (46 %)	56 (74 %)*
FEvG	75 (21 %)	42 (24 %)	10 (13 %)
Specific seizure pattern			
CS	141 (39 %)	53 (31 %)	42 (54 %)*
SE	71 (20 %)	31 (18 %)	22 (29 %)
AEDs used at first presentation			
None	180 (51 %)	99 (58 %)	28 (37 %)
Monotherapy	137 (38 %)	55 (32 %)	38 (50 %)
Polytherapy	41 (11 %)	18 (10 %)	10 (13 %)
Using other treatment			
No	281 (78 %)	159 (92 %)	30 (41 %)
Yes	77 (22 %)	13 (8 %)	46 (59 %)

*IdE* idiopathic epilepsy, *StE* structural epilepsy, *MR* magnetic resonance, *sz* seizure, *FES* focal epileptic seizure, *GES* generalized epileptic seizure, *FEvG* focal epileptic seizure evolving into generalized seizures, *CS* cluster seizures, *SE* status epilepticus, *AED* antiepileptic drug. *P < 0.05 (chi-squared test)

**Table 2:** Number and breed distribution of dogs with epilepsy, idiopathic, and structural epilepsy

Epilepsy (*n* = 358)		IdE (*n* = 172)		StE (*n* = 76)	
Breed	*n*	Breed	*n*	Breed	*n*
Chihuahua	54	Chihuahua	27	Chihuahua	15
Miniature Dachshund	36	Miniature Dachshund	18	Mixed-breed	7
Mixed-breed	28	Yorkshire Terrier	14	Miniature Dachshund	5
Yorkshire Terrier	24	Toy Poodle	13	Toy Poodle	
Toy Poodle	23	Labrador Retriever	10	Pug	
Labrador Retriever	17	Mixed-breed	9	Shih Tzu	
Shih Tzu	15	Miniature Schnauzer	7	Golden Retriever	4
Pomeranian	14	CKCS		Papillon	
Pug	13	Welsh Corgi	6	Welsh Corgi	3
CKCS	12	Golden Retriever		Labrador Retriever	2
Welsh Corgi	12	Shetland Sheepdog		Pomeranian	
Golden Retriever	11	Pug	5	Boston Terrier	
Shetland Sheepdog	10	Pomeranian	4	French Bulldog	
Miniature Schnauzer	9	Papillon		Shiba Inu	
Papillon	8	Japanese Spitz		Petit Basset Griffon Vendeen	
Shiba Inu	7	American Cocker Spaniel	3	Yorkshire Terrier	1
French Bulldog	6	French Bulldog		Miniature Schnauzer	
Maltese	5	Shiba Inu		Shetland Sheepdog	
Beagle	4	Beagle		CKCS	
Boston Terrier		Boston Terrier	2	Beagle	
Japanese Spitz		Maltese		Boxer	
American Cocker Spaniel	3	Italian Greyhound		Pekingese	
Bulldog		Bulldog		West Highland White Terrier	
Cairn Terrier		Shih Tzu	1	Cairn Terrier	
Italian Greyhound		Irish Setter		Bernese Mountain Dog	
Pekingese	2	Sealyham Terrier		Maltese	
Petit Basset Griffon Vendeen		Siberian Husky			
Siberian Husky		Miniature Poodle			
Afghan Hound	1	Afghan Hound			
Australian Shepherd		English Cocker Spaniel			
Basset Hound		Volpino Nano Bianco			
Bernese Mountain Dog		Australian Shepherd			
Bichon Frise		Dalmatian			
Bolognese		Hokkaido Dog			
Bouvier Des Flandres		Wirehaired Fox Terrier			
Boxer					
Dalmatian					
English Cocker Spaniel					
English Springer Spaniel					
Hokkaido Dog					
Irish Setter					
Japanese Chin					
Miniature Poodle					
Miniature Wirehaired Dachshunds					
Newfoundland					
Polish Lowland Sheepdog					
Samoyed					
Scottish Terrier					
Sealyham Terrier					
Volpino Nano Bianco					
West Highland White Terrier					
Wirehaired Fox Terrier					

*IdE* idiopathic epilepsy, *StE* structural epilepsy, *CKCS* Cavalier King Charles Spaniel

Regarding adherence to the IVETF diagnostic criteria for IdE (i.e., three-tier confidence level), in the IdE group, the complete MDB blood test and urinalysis suggested by IVETF was performed in 29 dogs and 4 dogs, respectively. Only two dogs fulfilled the tier I confidence level criteria. Although MRI was performed for 60 dogs, only 13 received CSF analysis. Therefore, 13 dogs fulfilled the tier II confidence level criteria (except for fasting and postprandial bile acids) but had insufficient MDB for tier I. Additionally, EEG examination (i.e., tier III confidence level) was performed in four dogs.

According to IVETF classification, all dogs with IdE had epilepsy of unknown cause, as no genetic and/or familial analyses were performed. The breeds, Lagotto Romganolo (*LGI2* mutation) [18] and Belgian Shepherd (*ADAM23* mutation) [19], which exhibit genetic epilepsy, were not present in this study. However, 53 dogs (nine breeds) were from the breeds listed as having suspected genetic epilepsy in an IVETF report [7].

In the StE group, MRI was performed for 76 dogs and 13 received CSF analysis. There was a tendency to avoid CSF tapping in anomalous and neoplastic diseases due to suspected elevated intracranial pressure, and similarly, in inflammatory disease in small-breed dogs with Chiari-like malformations. The StE group included 28 dogs (36.9 %) with inflammatory disease (MUO), 22 (28.9 %) with neoplastic disease, 9 (11.8 %) with anomalous disease, 6 (7.9 %) with vascular disease, and 1 (1.3 %) with traumatic disease. The remaining 10 dogs (13.2 %) were not possible to classify because of coexisting lesions.

When comparing clinical and demographic features of the IdE and StE groups, presence of CS (IdE, 31 % vs. StE, 55 %; *P* = 0.0004), FES (IdE, 30 % vs. StE, 13 %; *P* = 0.009), GES (IdE, 46 % vs. StE, 74 %; *P* < 0.0001), and seizure frequency at the time of first presentation (*P* < 0.0001) were significantly different in incidence (Table 1). Body weight did not differ between the two groups (*P* = 0.36). Additionally, there was no significant difference in gender (*P* = 0.12), neuter status (*P* = 0.89), presence of SE (*P* = 0.08), FEvG (*P* = 0.07), and multiple AED use (*P* = 0.69).

### Survival study

Of the 358 dogs with epilepsy that were included in the etiological study, we received 133 replies, of which 100 cases (consisting of 65 dogs with IdE and 35 dogs with StE) satisfied our survival analysis criteria. The results for each category of the survival study are described below.

#### All epilepsy cases (idiopathic epilepsy and structural epilepsy)

Of the 100 dogs with epilepsy, 51 dogs were alive at the end of the study period (March 2014), while 49 had died. Median lifespan was 13.0 years (156.6 months; 95 % CI, 131.1–181.6 months) and median survival time was 10.1 years (120.9 months; 95 % CI, 88.1–136.1 months).

#### Lifespan and survival time of dogs with idiopathic epilepsy

**Fig. 1:**
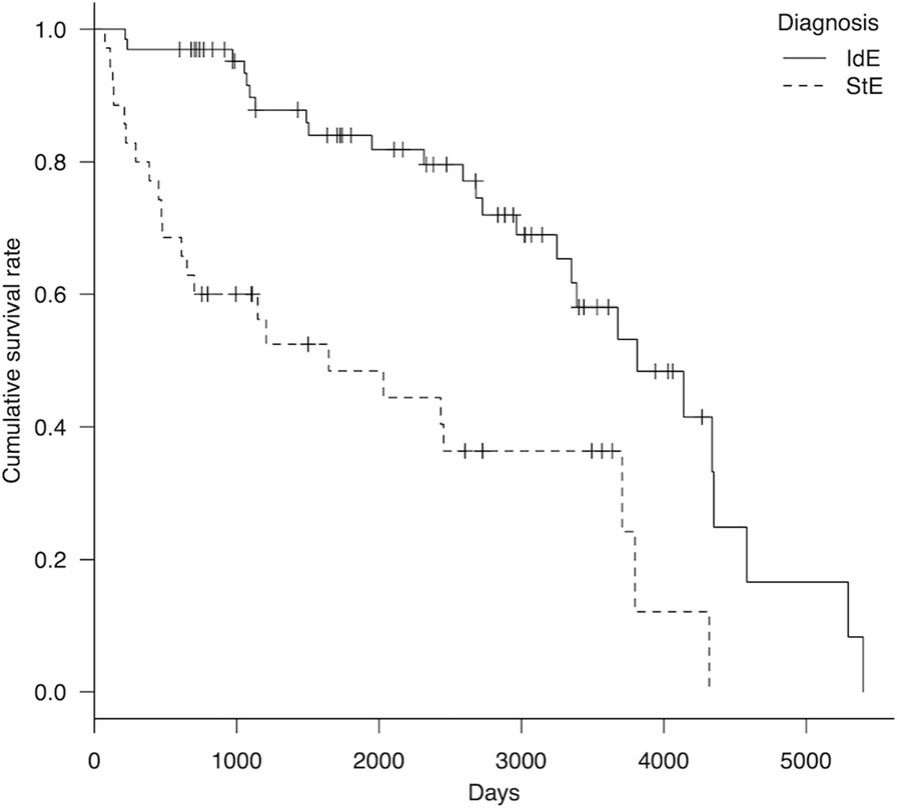
Kaplan–Meier curve of survival time in dogs with idiopathic and structural epilepsy. Survival time between the groups was significant (*P* = 0.00003). *Hash marks* indicate censored data. *IdE* idiopathic epilepsy, *StE* structural epilepsy

**Fig. 2:**
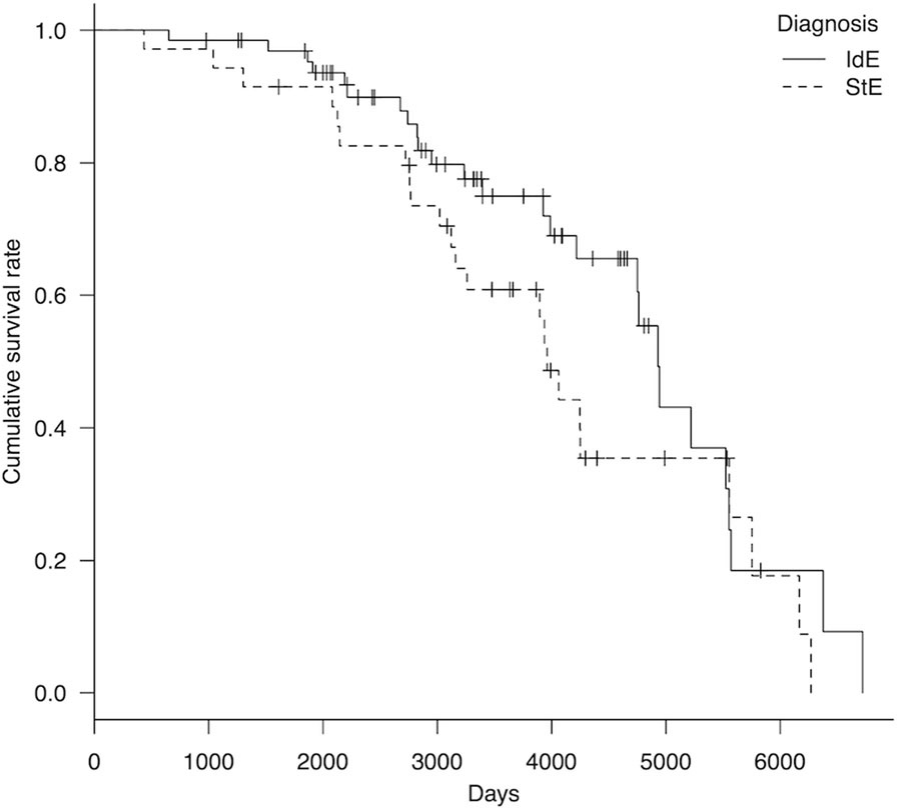
Kaplan–Meier curve of lifespan in dogs with idiopathic and structural epilepsy. There was no significant difference in lifespan between the two groups (*P* = 0.11). *Hash marks* indicate censored data. *IdE* idiopathic epilepsy, *StE* structural epilepsy

At the end of the study period (March 2014), 39 dogs were alive and 26 had died. Median lifespan and survival time were 13.5 years (162.0 months; 95 % CI, 138.6–182.5 months) and 10.4 years (125.4 months; 95 % CI, 106.8–143.0 months), respectively. Kaplan–Meier curves of survival time and lifespan for IdE are shown (Figs. 1 and 2, respectively).

There were no cases of euthanasia, and two dogs were thought to have died because of sudden unexpected death in epilepsy (SUDEP) after an epileptic seizure. These dogs were a Boston Terrier and a mixed-breed dog, and were 6.1 and 7.3 years old at the time of death, respectively. Both dogs had high seizure frequencies (≥4 sz/month), despite AED treatment using zonisamide and phenobarbital-potassium bromide, respectively. The Boston Terrier became dyspneic after a single FEvG and subsequently died, while the mixed-breed dog died suddenly after a single GES.

#### Lifespan and survival time of dogs with structural epilepsy

At the end of the study period (March 2014), 12 dogs were alive and 23 had died. Of those that died, 7 dogs (including 4 euthanized cases) had died because of uncontrolled epileptic seizures, 3 had died because of other diseases (e.g., intraoral melanoma and degenerative myelopathy), and 13 had died for unknown reasons. Median lifespan was 10.9 years (130.2 months; 95 % CI, 102.6–182.6 months) and median survival time was 4.5 years (54.1 months; 95 % CI, 20.1–121.9 months). Kaplan–Meier curves of survival time and lifespan are shown (Figs. 1 and 2, respectively).

**Fig. 3:**
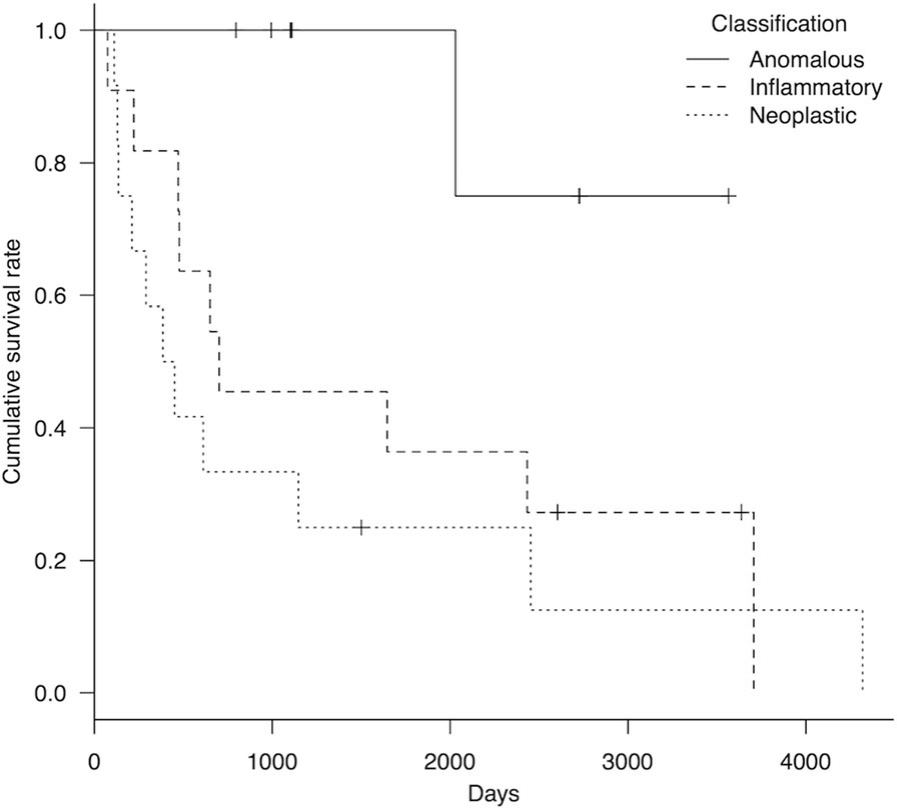
Kaplan–Meier curve of survival time in dogs with structural epilepsy caused by anomalous/inflammatory/neoplastic disease. Survival time among the three groups was significant (*P* = 0.02). *Hash marks* indicate censored data

**Fig. 4:**
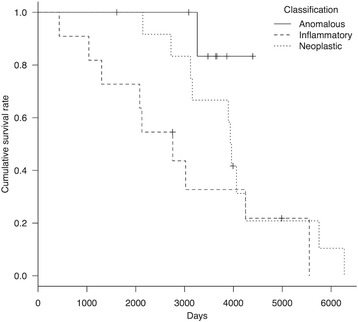
Kaplan–Meier curve of lifespan in dogs with structural epilepsy caused by anomalous/inflammatory/neoplastic disease. There was no significant difference in lifespan among the three groups (*P* = 0.07). *Hash marks* indicate censored data

Detailed sub-classification information is summarized in Table 3. There were no cases of infectious meningoencephalitis, therefore all inflammatory disease cases were clinically diagnosed as MUO. In addition to AED therapy, immunosuppressive treatments consisted of glucocorticoid and cyclosporine, and were administered to all dogs with MUO. Of 12 dogs with neoplastic disease, 2 had surgery and histopathological diagnoses of meningioma and osteosarcoma. Additional radiation therapy was performed in the dog with osteosarcoma. All dogs with neoplastic disease were treated using AEDs and decompression medications (e.g., glucocorticoid, glycerin, mannitol). Hydrocephalus was the most common anomalous disease (*n* = 4); these dogs received decompression medications as well as AED therapy (there were no surgical cases). The other anomalous diseases were polymicrogyria (*n* = 1), porencephaly (*n* = 1), diverticulum (*n* = 1), and morphological aberration of the olfactory bulb (*n* = 1); these cases were treated with AEDs and symptomatic therapy. Similarly, dogs with vascular disease received AEDs and symptomatic treatment. There were significant differences in survival time (*P* < 0.05) among the three sub-classifications (anomalous, inflammatory, and neoplastic disease), while there was no significant difference in lifespan. Kaplan–Meier curves for each group are shown (Figs. 3 and 4).

**Table 3:** Survival outcome of dogs with structural epilepsy by sub-classification

Variables		Survival time (years)		Lifespan (years)	
	*n* (dead)	Median	95 % CI	Median	95 % CI
Neoplastic	12 (11)	1.1	0.3–6.7	10.8	7.5–11.6
Inflammatory	11 (9)	4.5	1.3–∞	8.2	3.6–11.6
Anomalous	8 (1)	NA	5.6–∞	NA	8.9–∞
Vascular	2 (1)	10.4	∞–∞	16.9	∞–∞
Unclassified	2 (1)	3.3	3.3–∞	7.6	7.6–∞

*95 % CI* 95 % confidence interval, *NA* not available

Comparing median survival time between IdE and StE groups, dogs with IdE had a significantly longer survival time than dogs with StE (*P* < 0.001) (Fig. 1). However, there was no significant difference in lifespan between these two groups (Fig. 2).

#### Risk factors for survival time analysis

Of the 100 dogs with epilepsy in the survival analysis, six (including one with IdE and five with StE) were removed from the analysis of risk factors for survival time because of insufficient responses to the questionnaire. Clinical data from medical records and questionnaire responses are provided in Table 4, with the breed distribution in each category provided in Table 5. AED treatment choices during the clinical course in each group were obtained from questionnaire responses, and are shown in Table 6.

**Table 4:** Clinical data for dogs with idiopathic and structural epilepsy included in the multivariable analysis

Variables	Number of dogs		
	All epilepsy cases (*n* = 94)	IdE (*n* = 64)	StE (*n* = 30)
Gender			
Male [Neuter]	52 (55 %) [24 (26 %)]	37 (58 %) [18 (28 %)]	15 (50 %) [6 (20 %)]
Female [Neuter]	42 (45 %) [17 (18 %)]	27 (42 %) [11 (17 %)]	15 (50 %) [6 (20 %)]
Body weight (kg)			
Median	6.6	7.0	6.1
Range	1.3–35.0	1.3–31.0	2.0–35.0
Age at initial seizure onset (years)			
Median	3.1	2.7	6.0
Range	0.4–10.0	0.6–6.8	0.4–10.0
Neurological			
Normal or unremarkable	71 (76 %)	62 (97 %)	9 (30 %)
Deficits	23 (24 %)	2 (3 %)	21 (70 %)
Seizure frequency at last follow-up (sz/month)			
Median	3.3	2.5	4.0
Range	0.1–20.0	0.1–20.0	0.1–10.0
Seizure type			
FES	20 (21 %)	15 (23 %)	5 (17 %)
GES	51 (55 %)	30 (47 %)	21 (70 %)
FEvG	23 (24 %)	19 (30 %)	4 (13 %)
Specific seizure pattern			
CS	43 (46 %)	26 (41 %)	17 (57 %)
SE	16 (17 %)	13 (20 %)	3 (10 %)
Using AEDs at last follow-up			
None	46 (49 %)	37 (58 %)	11 (37 %)
Monotherapy	35 (37 %)	18 (28 %)	10 (33 %)
Polytherapy	13 (14 %)	9 (14 %)	9 (30 %)
Using other treatment			
No	69 (73 %)	60 (94 %)	9 (30 %)
Yes	25 (27 %)	4 (6 %)	21 (70 %)

*IdE* idiopathic epilepsy, *StE* structural epilepsy, *MR* magnetic resonance, *sz* seizure, *FES* focal epileptic seizure, *GES* generalized epileptic seizure, *FEvG* focal epileptic seizure evolving into generalized epileptic seizures, *CS* cluster seizures, *SE* status epilepticus, *AED* antiepileptic drug

**Table 5:** Number and breed distribution of dogs with idiopathic/structural epilepsy included in the multivariable analysis

IdE (*n* = 64)		StE (*n* = 30)	
Breed	*n*	Breed	*n*
Chihuahua	8	Chihuahua	5
Toy Poodle	6	Miniature Dachshund	3
Mixed-breed	5	Mixed-breed	
Yorkshire Terrier		Welsh Corgi	
Labrador Retriever	4	Labrador Retriever	2
Welsh Corgi		Papillon	
Golden Retriever	3	Pug	
Miniature Dachshund		Shih Tzu	
Miniature Schnauzer		Boston Terrier	1
American Cocker Spaniel	2	Boxer	
Boston Terrier		Golden Retriever	
CKCS		French Bulldog	
Japanese Spitz		Maltese	
Pomeranian		Miniature Schnauzer	
Pug		Petit Basset Griffon Vendeen	
Shetland Sheepdog		Pomeranian	
Beagle	1		
French Bulldog			
Hokkaido Dog			
Irish Setter			
Maltese			
Papillon			
Sealyham Terrier			
Shiba Inu			
Siberian Husky			

*IdE* idiopathic epilepsy, *StE* structural epilepsy, *CKCS* Cavalier King Charles Spaniel

**Table 6:** Antiepileptic drug choices in the multivariable analysis

Choice of AEDs	IdE (*n* = 64)	StE (*n* = 30)
	*n*	*n*
PB	9	9
ZNS	8	4
KBr	1	1
DZP	0	1
PB + ZNS	2	0
PB + KBr	5	3
ZNS + KBr	10	2
ZNS + DZP	1	0
PB + KBr + ZNS	8	0
PB + KBr + GBP	0	1
PB + ZNS + CZP	1	0
PB + KBr + ZNS + GBP	0	1
PB + KBr + ZNS + LEV	0	1
No AEDs	19	7

*AEDs* antiepileptic drugs, *IdE* idiopathic epilepsy, *StE* structural epilepsy, *PB* phenobarbital, *ZNS* zonisamide, *KBr* potassium bromide, *DZP* diazepam, *GBP* gabapentin, *CZP* clonazepam, *LEV* levetiracetam

Log-rank test results are shown in Table 7. In all epilepsy cases and IdE, the survival time of dogs with a seizure frequency of ≥0.3 sz/month was significantly shorter compared with those with frequency <0.3 sz/month. Cox proportional hazards for multivariable analysis showed that a seizure frequency of ≥0.3 sz/month (*P* = 0.00005, HR, 5.26, 95 % CI 2.37–11.70) in the all epilepsy group, and a seizure frequency of ≥0.3 sz/month (*P* = 0.0005, HR, 9.80, 95 % CI 2.70–35.52) and FES (*P* = 0.04, HR, 3.99, 95 % CI 1.05–15.17) in the IdE group were significantly negatively correlated with survival time. No significant differences were detected with the other risk factors in all epilepsy group and IdE.

**Table 7:** Log-rank test results for dogs with idiopathic and structural epilepsy

Variables	All epilepsy cases (years)			IdE (years)			StE (years)		
	*n* (dead)	median (95 % CI)	*P* value	*n* (dead)	median (95 % CI)	*P* value	*n* (dead)	median (95 % CI)	*P* value
Gender									
Male	52 (30)	8.9 (5.6–11.8)	0.26	37 (18)	11.3 (7.3–12.6)	0.45	15 (12)	4.5 (0.4–10.4)	0.20
Female	42 (13)	10.1 (9.2–∞)		27 (7)	10.1 (9.2–∞)		15 (6)	NA (1.9–∞)	
Status of neuter									
Intact	53 (26)	10.2 (8.1–11.8)	0.31	35 (15)	10.4 (8.9–14.5)	0.31	18 (11)	10.2 (1.3–∞)	0.46
Neuter	41 (17)	9.3 (6.3–∞)		29 (10)	9.3 (6.3–∞)		12 (7)	6.7 (0.4–∞)	
Body weight									
< 6.6 kg	47 (20)	10.2 (6.7–14.5)	0.26	31 (11)	11.9 (7.5–∞)	0.28	16 (9)	6.7 (3.3–∞)	0.27
≥ 6.6 kg	47 (23)	9.3 (7.1–10.4)		33 (14)	10.1 (7.3–∞)		14 (9)	4.0 (0.4–∞)	
Seizure frequency at last follow-up									
< 0.3 sz/month	48 (16)	11.8 (10.4–14.5)	<0.01	37 (11)	11.9 (11.3–14.5)	<0.01	11 (5)	10.4 (0.58–∞)	0.06
≥ 0.3 sz/month	46 (27)	7.3 (4.5–9.2)		27 (14)	8.9 (5.3–10.1)		19 (13)	4.5 (1.2–∞)	
Epileptic seizure type									
FES	20 (6)	8.9 (3.3–∞)	0.77	15 (4)	8.9 (2.9–∞)	0.20	5 (2)	NA (0.2–∞)	0.89
GES	51 (26)	10.4 (7.3–11.9)		30 (12)	11.3 (10.1–14.5)		21 (14)	5.6 (1.2–10.4)	
FEvG	23 (11)	8.1 (6.3–∞)		19 (9)	9.2 (5.3–∞)		4 (2)	6.7 (6.7–∞)	
Development of CS									
Yes	43 (22)	9.2 (4.1–11.3)	0.06	26 (11)	10.4 (4.1–∞)	0.21	17 (11)	4.5 (1.1–∞)	0.18
No	51 (21)	10.1 (8.1–11.9)		38 (14)	10.1 (8.1–14.5)		13 (7)	6.7 (3.3–∞)	
Development of SE									
Yes	16 (9)	10.1 (4.1–11.9)	0.93	13 (7)	10.1 (4.1–∞)	0.42	3 (2)	10.4 (3.3–∞)	0.54
No	78 (34)	10.2 (7.5–11.8)		51 (18)	10.4 (8.9–∞)		27 (16)	6.7 (1.3–∞)	
Using AEDs at last follow-up									
None or monotherapy	58 (26)	10.4 (7.1–12.6)	0.48	37 (14)	11.9 (7.5–14.5)	0.29	22 (12)	5.6 (1.3–∞)	0.50
Polytherapy	36 (17)	9.3 (6.7–10.4)		27 (11)	9.3 (7.3–11.3)		8 (6)	6.7 (1.1–∞)	

*IdE* idiopathic epilepsy, *StE* structural epilepsy, *95 % CI* 95 % confidence interval, *GES* generalized epileptic seizure, *FES* focal epileptic seizure, *sz* seizure, *FEvG* focal epileptic seizure evolving into generalized epileptic seizures, *CS* cluster seizures, *SE* epilepticus status, *AED* antiepileptic drug, *NA* not available

## Discussion

This study is the first report on the etiology of canine epilepsy using the IVETF classification of 2015. We found that the prevalence of epilepsy was approximately 1.9 % in all dogs that presented to the teaching hospital (referrals only), and 0.9 and 0.4 % for IdE and StE, respectively. These percentages are similar to previous studies reporting 1–2.6 % for epilepsy in veterinary referral clinics, and 0.5–5 % for IdE in primary clinics [7]. These findings suggest that differences in breeds or countries are not associated with prevalence of epilepsy.

Median survival time in the IdE group (10.4 years) was longer than in the StE group (4.5 years), which is supported by previous studies (IdE, 9.2 years and StE, 5.8 years [12]; IdE, 10.5 years and StE, 3.4 years [20]). Additionally, median lifespan in all epilepsy cases (IdE and StE), IdE, and StE were 13.0, 13.5, and 10.9 years, respectively. Previous studies reported median lifespans in dogs with epilepsy, including all sub-types (i.e., IdE, StE, and unknown cause), of 7.6 years [12] and 7.0 years [21], which are clearly exceeded by the present study.

A reason for this discrepancy may be the number of euthanized dogs: in our study, only 4/100 dogs were euthanized for epilepsy-related causes, whereas 49/81 [12] and 24/63 [21] dogs were euthanized in the previous studies. Moreover, all euthanized dogs in the present study were StE cases. In the previous study, it was suggested that euthanasia was chosen in many cases because of quality of life (QOL) degradation due to uncontrolled epileptic seizures [22]. Certainly, in our study, euthanized dogs appeared to have a lower QOL because of uncontrolled seizures. However, the owners (especially those of dogs with IdE) had a tendency to refuse euthanasia (and also necropsy) because euthanasia is regarded negatively by the majority of Japanese people.

Another possibility is that companion dogs in Japan have longer lifespans than those in Western countries. Inoue et al. (2015) reported a life expectancy of 13.7 years for dogs in Japan [23]. Our study shows that the median lifespan of dogs with IdE is as long as companion dogs in Japan. In contrast, previous studies reported that the median lifespan in companion dogs in Denmark [24] and median longevity in England [25] were 10.0 and 12.0 years, respectively. The breed population of general companion dogs and/or dogs with epilepsy in Denmark included a great number of large-breed dogs such as German Shepherd and Labrador Retriever. Our present study (and also the previously cited Japanese data [23]) is composed of many small-breed dogs such as Chihuahua, Toy Poodle, and Miniature Dachshund, reflecting the current feature of breed populations in Japan. In general, the lifespan of large-breed dogs is shorter than small-breed dogs [26]. Therefore, this difference in breed populations between Western countries and Japan may also influence the lifespan of dogs with epilepsy.

Regarding risk factors for survival time in IdE, the presence of FES was found to be significantly associated with reduced survival time in the present study; however, this has not been found in previous studies [21]. Recently, Packer et al. (2015) reported that when classifying seizures into seizure types, FES was the least agreed upon classification between veterinarians and neurology specialists [27]. Therefore, it appears difficult to detect FES in animals for veterinarians, let alone for owners. Furthermore, in experimental studies of kindling and/or kainic acid, recurrent FES induces more severe seizure frequency (i.e., kindling phenomenon) and seizure pattern (e.g., CS, SE, and/or secondary generalizations), which results in neuronal loss and/or secondary epileptogenesis such as mirror focus [28–31]. Thus, we urge practitioners not to think of FES as inferior to GES, and FES should be treated appropriately even if the apparent symptoms are short or subtle. In this regard, EEG may be useful to differentiate FES and GES, although EEG use in veterinary medicine is not common, and its availability and standardized methodology have not yet been well established.

Additionally, we found that a seizure frequency of ≥0.3 sz/month was a risk factor for reduced survival time in IdE. Death or euthanasia in dogs with epilepsy is associated with unacceptable epileptic seizure control, which shortens survival time compared with other causes [21]. As mentioned above, no case with IdE was euthanized in our study. Therefore, our result indicates that a risk factor for reduced survival time in IdE includes seizure frequency (≥0.3 sz/month), regardless of euthanasia. In addition, we showed that use of multiple AEDs (polytherapy) was not a risk factor affecting survival time in IdE. The IVETF proposal states that the effect of drug-resistant epilepsy on outcome is not precisely understood [9]. Furthermore, some studies [12, 32] have shown that survival of dogs receiving multiple AED treatments does not differ significantly from that of dogs receiving single AED treatment (monotherapy). These results suggest that multiple AED treatment may not directly influence survival time. Therefore, we assume that a high seizure frequency (≥0.3 sz/month) is a risk factor for reduced survival time regardless of whether dogs with IdE receive polytherapy.

In our study, two dogs were thought to have died due to SUDEP. A few studies have reported SUDEP in veterinary medicine [33, 34], but its mechanisms remain unknown. In humans, patients at highest risk for SUDEP show occurrence of generalized tonic-clonic seizures, poor seizure control, a young age, and multiple AED treatment [35]. Here, the two dogs with suspected SUDEP died at a relatively young age (6.1 and 7.3 years) and had a very high epileptic seizure frequency (≥1 sz/week) with a poor response to at least one AED treatment. Although there had only been very small numbers reported, the risk of SUDEP in dogs is proposed to be similar to humans. In addition, canine SUDEP was previously reported in an Akita-inu [33] and Labrador Retriever [34], while here it was a Boston Terrier and a mixed-breed dog. Indeed, it is likely that SUDEP may occur in various breeds.

In sub-classified StE, there was a significant difference in survival time among the three groups (anomalous, inflammatory, and neoplastic disease). The log-rank test and Kaplan–Meier method showed that >80 % of dogs with anomalous disease were alive over 5 years after the initial epileptic seizure onset. Consequently, if a suitable treatment (including AED therapy) is acceptable, StE due to anomalous disease might not be a risk factor for reduced survival time, compared with neoplastic and inflammatory disease. Nevertheless, survival time is variable depending on the type or severity of malformation as well as seizure severity. In contrast, median survival time in neoplastic and inflammatory disease was 13.7 and 54.1 months, respectively. Previously, median survival time in dogs with neoplastic disease that received symptomatic treatment, and inflammatory disease that received immunosuppressive treatment, were reported as approximately 2.2–23.3 months [35] and 77.5 months [36], respectively. Therefore, it is important for survival of dogs with StE to recognize sub-classification by signalment, clinical course, and MRI/CSF findings. Because the StE group was composed of various diseases and had some limitations as described below, we did not perform risk factor analysis for survival time in StE cases. However, a seizure frequency of ≥0.3 sz/month was found to be a risk factor for reduced survival time in all epilepsy cases, including StE. This may suggest that seizure control is also important for survival in StE, as well as specific therapies for causative disease.

We observed significant differences in prevalence of FES, GES, and CS between the IdE and StE groups. A previous study stated that it is not possible to differentiate between IdE and StE based on seizure type, but that FES is more suggestive of StE [37]. FES is frequently caused by neoplastic disease, which is probably due to the lesion location being mainly single and focal. Nevertheless, we observed significantly more FES in the IdE group (30 %) than the StE group (13 %). Additionally, breeds in our StE group were different (i.e., the majority were small-breed dogs, as mentioned above) compared with previous reports [37–39], which included many large dog breeds such as Boxer, Labrador Retriever, and German Shepherd. In particular, 74.2 % of Boxers presenting with epileptic seizures had asymmetrical brain lesions, and many cases were associated with neoplastic disease [39]. In previous studies, the prevalence of neoplastic and inflammatory disease in StE was 22–72 and 7–37 %, respectively [37, 38, 40, 41]. In contrast, in our study, StE included many small dog breeds such as Chihuahua, Miniature Dachshund, Toy Poodle, Pug, and Shih Tzu, which show a predisposition to MUO. Furthermore, only one Boxer was included in our study. Consequently, prevalence of neoplastic and inflammatory disease in our study was 28.2 and 35.9 %, respectively. A previous study reported that the major epileptic seizure type in dogs with inflammatory disease is GES [42]. Because many MUO cases implicate multifocal pathological lesions, GES (or FEvG) may frequently develop compared with FES. Therefore, it is difficult to use seizure type to diagnose StE in all cases, and it is necessary to investigate prevalence in various countries because breeds vary according to community. In contrast, we observed significantly more CS development in the StE group (55 %) compared with the IdE group (31 %). This is similar to a previous study (IdE, 45.2 % vs. StE, 65.6 % [34]). The IVETF recommends that MRI and CSF analysis are performed in dogs with CS at epileptic seizure onset [6]. Our result supports this recommendation.

There were some limitations to our study. Because this was a retrospective study, blood tests, urinalysis, genetic and/or familial analysis according to the IVETF consensus [6] were not fulfilled in most dogs with IdE. Furthermore, MRI in our study did not correspond to the epilepsy-specific MRI protocol suggested by IVETF [10]. Although we made efforts to follow the IVETF consensus as much as possible, we had to exclude 110 dogs with epilepsy that did not satisfy the criteria e.g., our initial cohort included many dogs that had an initial epileptic seizure onset at <6 months or >6 years and/or showed neurological deficits but were not examined by MRI or CSF analysis due to various reasons such as complications in the dogs and economic compliance of owners. Of course the IVETF consensus aims to standardize future worldwide studies, and researchers and practitioners should follow its proposals; however, it may be difficult for a retrospective study to completely comply with IVETF criteria. Additionally, because histopathological examination was not performed in most dogs with StE, its sub-classification was evaluated by signalment, clinical course, and MRI/CSF findings. Therefore, our etiological and survival study of dogs with StE may have not been accurately evaluated. Furthermore, the StE group in the present study was a very heterogeneous group with multiple diseases, and had a small number of cases in each disease category. Therefore, statistical power to detect significant association was thought to be low. The risk factors for survival time in StE may be found in a larger study, or a more homogeneous population.

## Conclusions

Our study indicates that dogs with well controlled IdE will survive as long as the lifespan of general pet dogs in Japan. In contrast to previous studies from Western countries, many small-breed dogs were included in our study and may have contributed to our findings of a long survival time and lifespan. However, we identified a high seizure frequency (>0.3 sz/month) and FES as risk factors for survival time in dogs with IdE. Furthermore, our study shows that survival time of dogs with StE is shorter than that of dogs with IdE. In particular, StE due to neoplastic and inflammatory disease is associated with a short survival time. In contrast to Western countries, our Japanese study found that inflammatory disease is more prevalent than neoplastic disease. If dogs exhibit onset of their initial epileptic seizure at a young to middle age (i.e., similar to IdE), it is important to differentiate between StE by inflammatory disease and IdE, especially within regions where small-breed dogs are popular.

## Abbreviations

AED: Antiepileptic drug
CBC: Complete blood count
CI: Confidence interval
CS: Cluster seizures
CSF: Cerebrospinal fluid
FES: Focal epileptic seizure
FEvG: Focal epileptic seizure evolving into generalized epileptic seizures
FLAIR: Fluid-attenuated inversion recovery
GES: Generalized epileptic seizure
HR: Hazard ratio
IdE: Idiopathic epilepsy
IVETF: International Veterinary Epilepsy Task Force
MRI: Magnetic resonance imaging
MUO: Meningoencephalitis of unknown origin
QOL: Quality of life
SE: Status epilepticus
StE: Structural epilepsy
SUDEP: Sudden unexpected death in epilepsy
sz: Seizures
